# Broken Instrument Removal from Mandibular First Molar with Cone-beam Computed Tomography based Pre-operative Computer-assisted Simulation: A Case Report

**DOI:** 10.31729/jnma.5231

**Published:** 2021-08-31

**Authors:** Babita Pradhan, Yuan Gao, Yuan Gao, Tingwei Guo, Yangpei Cao, Jinzhi He

**Affiliations:** 1 State Key Laboratory of Oral Diseases, Department of Cariology and Endodontics, West China Hospital of Stomatology, Sichuan University, Chengdu, China; 2 Center for Craniofacial Molecular Biology, Herman Ostrow School of Dentistry, University of Southern California, 2250 Alcazar Street, Los Angeles, CA 90033, USA

**Keywords:** *cone-beam computed tomography*, *root canal therapy*, *treatment outcome*

## Abstract

Intracanal separation of nickel titanium files hinders complete shaping, cleaning, and filling of the root canal system and ultimately influences the endodontic treatment outcome. In this case report, we presented a successful broken instrument retrieval from the middle third of the mesiobuccal root canal of tooth #30 with the assistance of cone-beam computed tomography based preoperative computer-assisted simulation, micro-trepan bur and micro-tube from Micro-Retrieve & Repair system and dental operative microscope. The involved tooth was then successfully cleaned, shaped and obturated followed by coronal restoration. At the three-year follow-up, tooth #30 was asymptomatic and functioned well without radiographic changes. The present case provides an example to show the robustness of computer-assisted technology in dental procedures and to show how the combination of advanced techniques can facilitate root canal therapy.

## INTRODUCTION

Instrument separation of nickel titanium (Ni-Ti) files within the root canal is an unfortunate procedural accident. When it comes to fractured instrument removal, computer-assisted technology has its powerful potentials. To the best of our knowledge, the case reports published,^1-3^ has not shown the application of computer-assisted technology in fragment retrieval in vivo. In the present study, we presented a case report, in which the broken Ni-Ti instrument was removed from the mandibular right first molar with the aid of cone-beam computed tomography (CBCT) based preoperative computer-assisted simulation, Micro-Retrieve & Repair system as well as dental operating microscope (DOM).

## CASE REPORT

A 32-year-old male without contributory medical history was referred to the department of endodontics and the chief complaint was a broken instrument in lower right posterior teeth region. The patient's dental history indicated that there was a fractured instrument in the mandibular right posterior tooth for one week. Intraoral examination revealed that a temporary restoration was placed on tooth #30. The percussion test produced a slight response, and the patient complained of pain during the bite test. In addition, mobility of tooth #30 scored as 1° (less than 1mm), and periodontal probing depth was less than 3mm. No sinus tract was observed around tooth #30.

**Figure 1:**
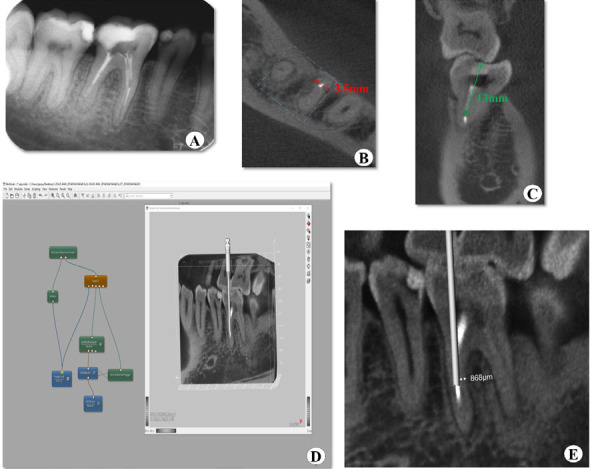
(A) Preoperative radiograph showing the broken instrument in the mesial canal. (B) Crossectional view of CBCT confirming broken instrument in the mesiobuccal root canal of tooth #30 and 3.5mm mesiodistal distance (red arrow). (C)Coronal view of the CBCT image indicating the distance between the open access and the coronal end of broken instrument (green arrow)=13mm. (D) Placement of a virtual micro-trepan bur around the broken instrument using Mevislab. (E) Remaining dentin thickness=868μm obtained.

The radiograph confirmed that tooth #30 had intracanal medicament and a separated endodontic instrument (approximately 3-4 mm in length) located at the middle third of the mesial root canal (Figure 1A). There was no periapical radiolucency surrounding the tooth. A CBCT scanning (3D Accuitomo; Morita, Kyoto, Japan) was scheduled to visualize the tooth anatomy and the broken instrument in a three dimensional (3D) view. The cross-sectional view of CBCT images clearly showed that the broken instrument was in the mesiobuccal canal of tooth #30 and the mesiodistal diameter of the involved root canal was ~3.5 mm at the site where the broken instrument was (Fig IB). The coronal view of the CBCT image further confirmed that the distance between the coronal end of the broken instrument to the access opening was ~13 mm (Figure 1C).

The preoperative computer-assisted simulation of instrument fragment removal was done using the MeVisLab package (MeVis Research, Bremen, Germany). The CBCT data was imported into Mevislab to build an environment to perform an interactively virtual canal path preparation, which simulates the process of broken instrument removal under actual condition. Similar to the previously published study,^4^ the following steps were included in the framework (Figure 1D):

1. A 3D data set from the scanned broken instrument model CBCT image was build.
2. A virtual micro-trepan bur (a cylinder) in the canal was created and the size for the micro-trepan (0.9 mm diameter according to the actual size) was set.
3. Then the virtual micro-trepan bur was interactively placed to a proper position around the broken instrument (1 mm apical to the coronal end) to simulate removing dentin to form a narrow parallel cylindrical space.
4. Then the image with the cylinder for subsequent remaining dentin thickness analysis and measurement was saved (Figure 1E). According to the simulation, the remaining dentin thickness was 868 μm.

Then micro-trepan bur and micro-tube for the removal of the broken instrument were planned, and the risk and prognosis were explained to the patient. Informed consent was obtained from the patient before the operation.

**Figure 2:**
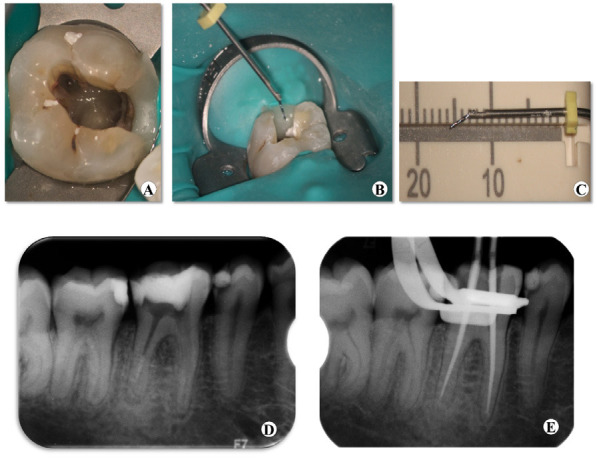
(A) After refining the access cavity and removal of intracanal medication the broken instrument could be observed in the mesiobuccal root canal under DOM. (B) Micro-tube used to retrieve the broken instrument from the mesiobuccal root canal. (C) Confirming the length of the broken instrument=3.5mm. (D) Radiograph confirming complete removal of the broken instrument. (E) Radiograph of the master cone gutta-percha.

The tooth was isolated using a rubber dam, and the endodontic access was refined with a long tapered diamond bur and under DOM (OPMI PROergo; Carl Zeiss, Oberkochen, Germany). After complete removal of calcium hydroxide intracanal medicament which was previously placed in the root canal by the former clinician, the broken instrument could be observed in the mesiobuccal canal with the help of the DOM (Figure 2A). In order to get a linear straight path to the most coronal portion of the broken instrument, coronal enlargement of the mesiobuccal canal was achieved by using Gates Glidden(GG) burs (nos. 1-3) (Dentsply Maillefer, Ballaigues, Switzerland). A staging platform as described by Ruddle,^5^ was prepared at the coronal end of the broken instrument using modified GG burs (no. 3) Then, a 20-G micro-trepan bur from Micro-Retrieve & Repair system with a 0.9-mm outside diameter and a 0.6-mm inside diameter was selected as per the simulation and operated with an endodontic motor (Dentsply Malliefer, Ballaigues, Switzerland) in a counterclockwise direction (500 rpm) to expose a ~1 mm length of the broken fragment by removing the surrounding dentin. After removing the dentin surrounding the broken instrument, the micro-tube was used to withdraw the fragment from the mesiobuccal canal (Figure 2B). The length of the broken fragment was ~3.5 mm (Figure 2C). Complete retrieval of the broken fragment from tooth #30 was double confirmed by a post-operative radiograph (Figure 2D). Then the root canals were copiously irrigated with 1% NaOCl using a 30-G side vented irrigation needle NaviTip (Ultradent Products, South Jordan, USA), and dried with paper points. All the root canals were filled with calcium hydroxide paste (ApexCal, Ivoclar Vivadent) and the cavity was sealed with temporary filling material Caviton (GC company, Japan). A second visit was scheduled one week later.

**Figure 3:**
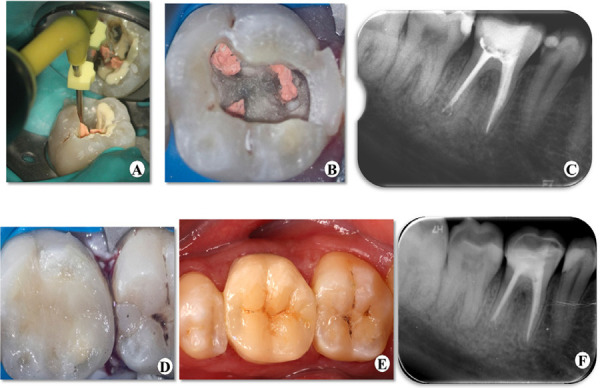
(A) A heated plugger-Alphall used to remove coronal portion of gutta-percha. (B) Pulp chamber floor showing the canal orifice after the obturation.(C) Post obturation radiograph showing the completely filled canal. (D) Postoperative clinical view of tooth #30 after restoration. (E) Occlusal view of the occlusal surface of tooth #30 with onlay. (F) Three-year-follow up radiograph showing no radiographic changes.

On the second visit, the patient was asymptomatic and the temporary filling was intact. The working length was confirmed using an apex locator (Root ZX; Morita, Tokyo, Japan) for all four root canals. The canals were prepared with ProTaper Universal rotary file system (Dentsply Malliefer, Ballaigues, Switzerland). After completion of chemo-mechanical preparation, the canals were irrigated with 1% NaOCl and dried with paper points. The master cone was fitted and confirmed by radiograph (Figure 2E). AH Plus Jet root canal sealer (Dentsply Maillefer, USA) was placed along with the gutta-percha. A heated plugger-Alpha II (B&L Biotech, Inc. Virginia, USA) was used to remove the coronal portion of the gutta-percha (Figure 3A) and the coronal canal space was back-filled using small preheated pieces of gutta-percha with a warm gutta-percha delivery system (Figure 3B). A postoperative radiograph was taken to assess the quality of obturation (Figure 3C). After a week of post obturation period, the patient was scheduled for post obturation restoration. (Figure 3D). Then the tooth #30 was rehabilitated with CAD-CAM ceramic onlay (Figure 3E). On the third year follow-up on tooth #30 was asymptomatic with no radiographic changes (Figure 3F) and functioned well.

## DISCUSSION

In the present case report, the micro-trepan/microtube technique was chosen to remove the broken instrument in both preoperative simulation and clinically in tooth #30. In previous studies,^4,6-8^ instrument removal procedures were simulated successfully, and the remaining dentin thickness was measured. In the present report, the framework used in Mevislab helped to perform advanced simulation and analysis including virtual canal preparation using the micro-trepan bur shape to simulate the dentin removal path around the broken instrument for the retrieval of the broken instrument and perform preoperative analysis of benefit and risk factors. Pre-operative computer-assisted simulation of broken instrument removal provided a powerful platform in measuring remaining dentin wall thickness in the present study. According to Lim and Stock,^9^ 200-300|jm dentin thickness should be retained after preparation in order to withstand compaction forces during obturation and to prevent perforation or fracture. Also, the attempt of retrieval may be risky if the value falls below a certain value. The remaining dentin thickness measured in the present report 868μm was greater than 200-300μm, thus it was relatively safe and significant to preserve tooth integrity.

Ultrasonics when used during the retrieval process to trough the coronal portion of dentin around the broken instrument then it leads to loss of more dentin surface when compared with micro-trepan bur.^10^ Secondly, even if the ultrasonics is able to trough the dentin around the coronal fragment it cannot be retrieved out on its own. In this scenario, micro-tube can be a helpful aid to retrieve the broken instrument out from the root canal. Thus the use of micro-trepan bur and micro-tube helps to save the dentin and preserve the integrity of the tooth. However, on the other hand, excess removal of dentin during trephine may result in perforation or vertical fracture. The clinician should perform a sufficient preoperative assessment of the root canal morphology and the location of broken fragment followed by selection of the removal technique which helps in the minimum removal of dentin preserving tooth integrity.

The fractured instrument especially a broken Ni-Ti file leads to obstruction in the root canal and blocks thorough cleaning and shaping procedures during the root canal therapy. In the context of broken instrument removal, location of the broken instrument, remaining dentin thickness, possible complications should be assessed prior thoroughly for proper decision making and treatment planning.

The present case report showed that combined with software, CBCT data can help to simulate the removal of broken instrument technique and also determine the remaining dentin thickness. Therefore, this type of preoperative assessment can be a safe and conservative method for better treatment outcome in broken instrument retrieval.
